# Supplementary figures and images for: Alveolar niche disruption and aberrant epithelial reprogramming are early hallmarks of idiopathic pulmonary fibrosis
Source: bioRxiv. 2026 May 30:2026.05.27.727792. Preprint. [Version 1] doi: 10.64898/2026.05.27.727792 (PMC13317612; doi:10.64898/2026.05.27.727792)

## Florence cohort

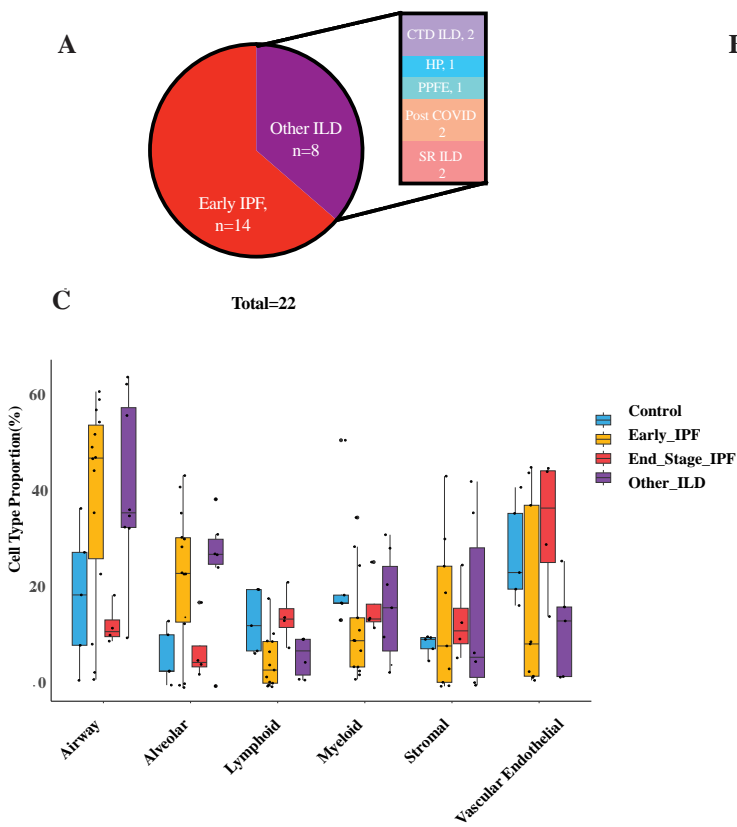

## NIH cohort, n=9

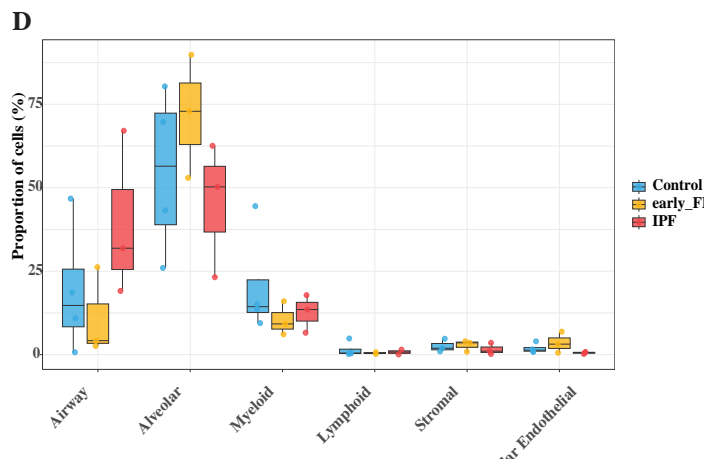

**E**

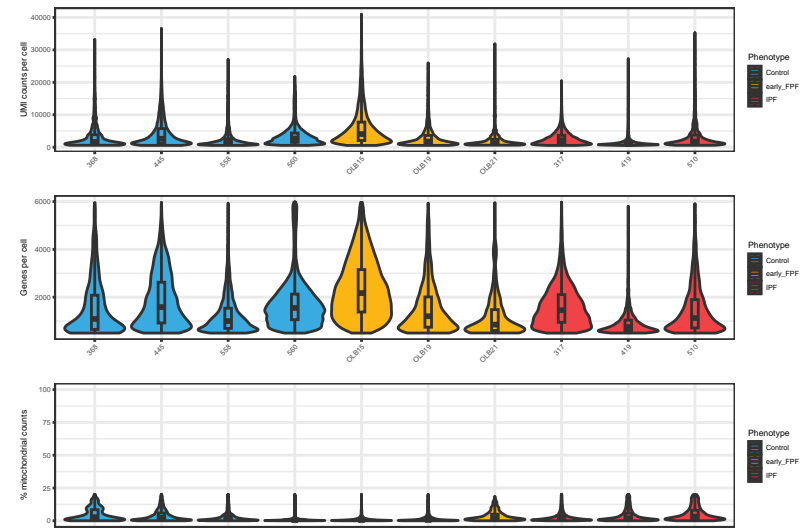

## Forli cohort

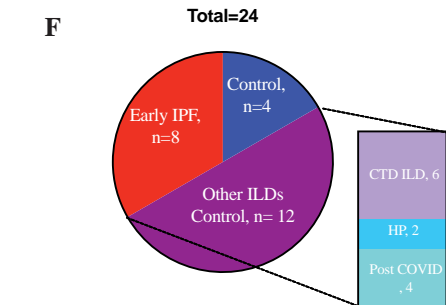

**G**

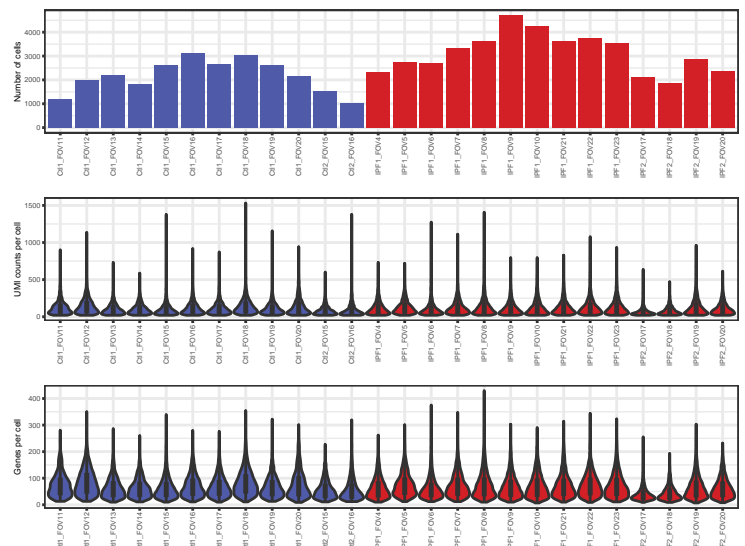

Supplement: Supplement 2 [file media-2.pdf]

A

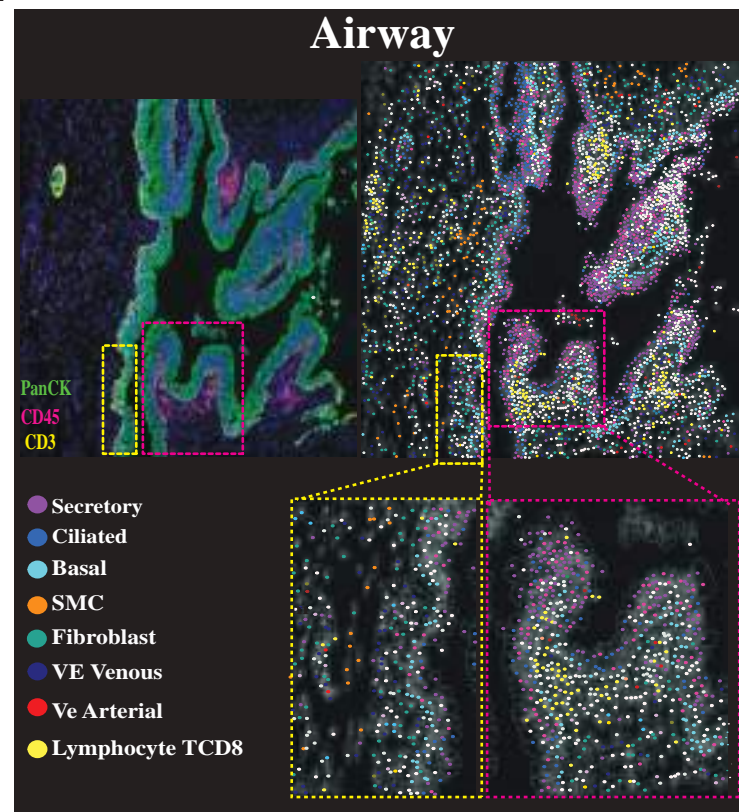

B

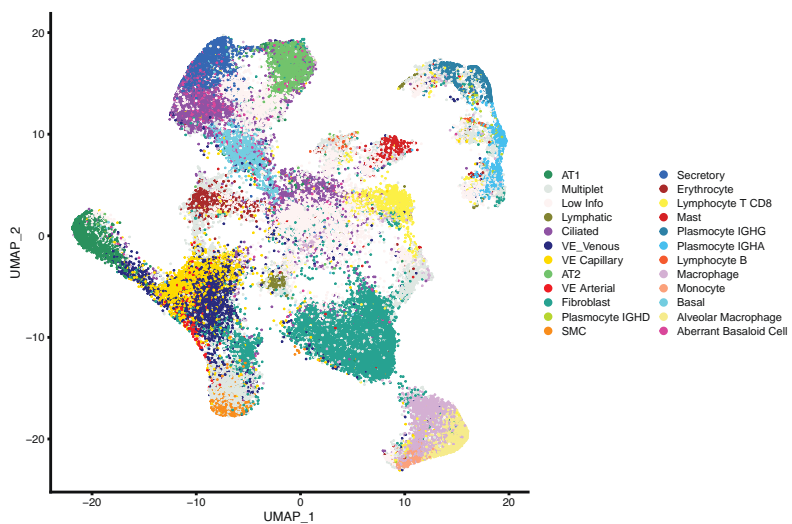

C

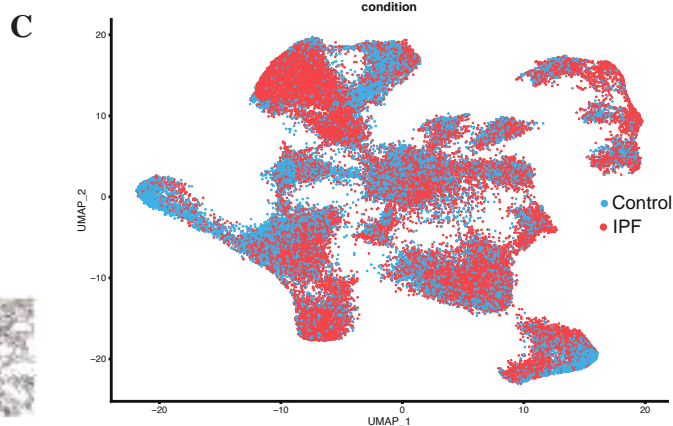

D

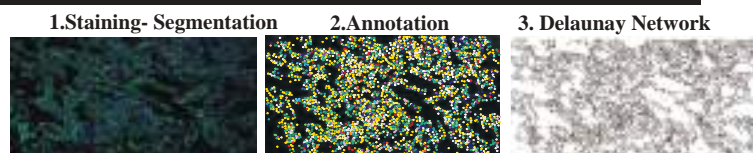

E

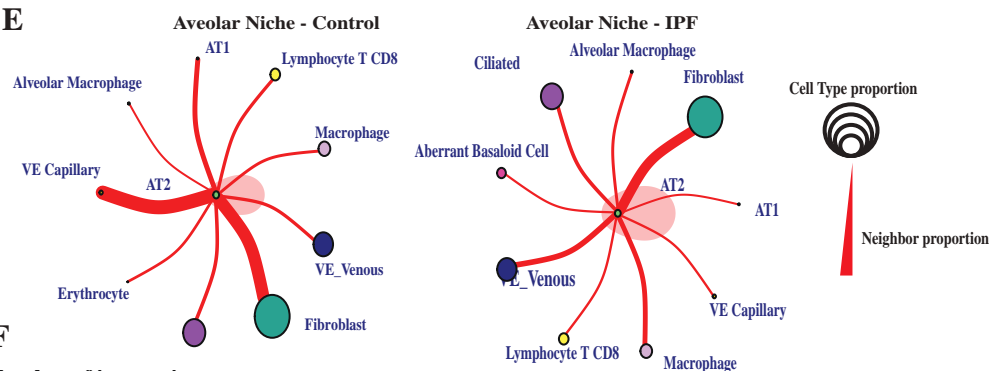

F

Number of interactions

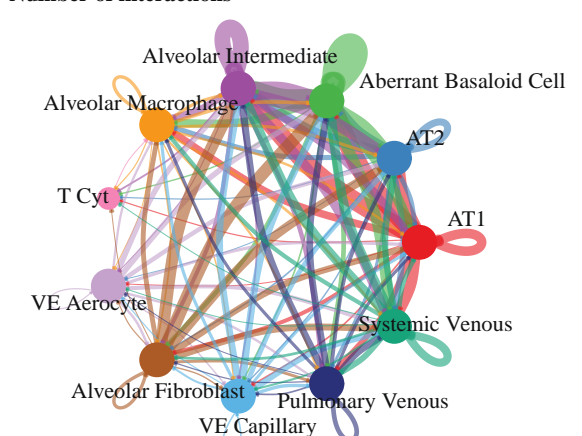

Interaction strength

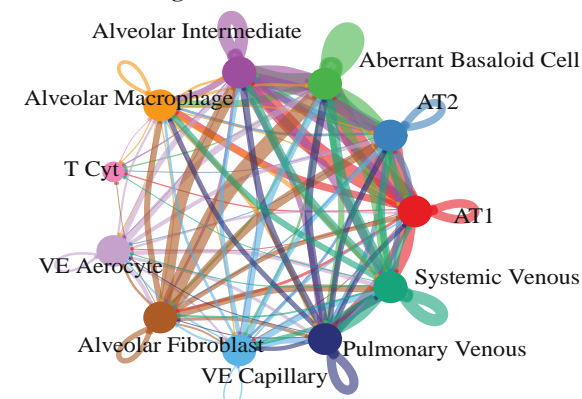

G

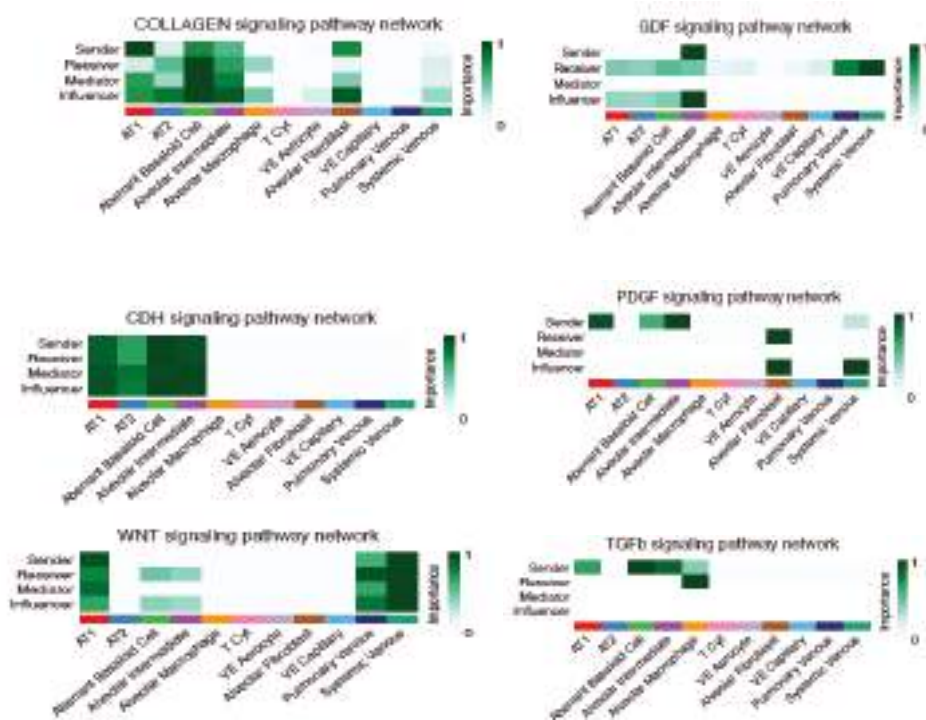

Supplement: Supplement 5 [file media-5.pdf]
