# Supplementary material for: Alveolar niche disruption and aberrant epithelial reprogramming are early hallmarks of idiopathic pulmonary fibrosis
Source: bioRxiv. 2026 May 30:2026.05.27.727792. Preprint. [Version 1] doi: 10.64898/2026.05.27.727792 (PMC13317612; doi:10.64898/2026.05.27.727792)
Supplement: Supplement 4 [file media-4.pdf]

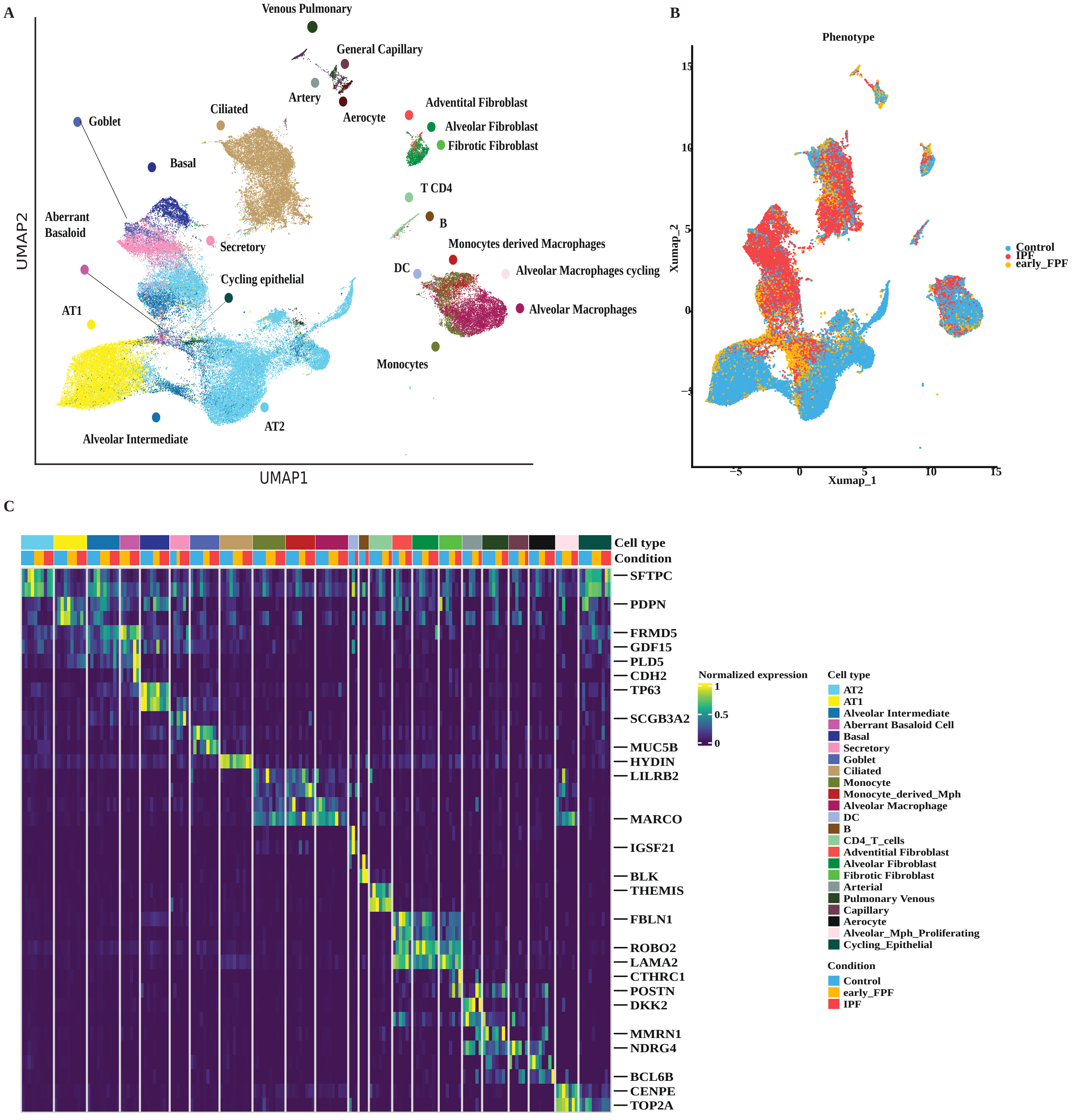

D

	AT1	AT2	Aberrant Basaloid Cell	Adventitial Fibroblast	Alveolar Fibroblast	Alveolar Intermediate	Alveolar Macrophage	B	Basal	Ciliated	DC	Aerocyte	Arterial	Capillary	Pulmonary Venous	Fibrotic Fibroblast	Goblet	Lymphatic	Mast	Monocyte	Secretory
AT1	0.97	0.09	0.25	-0.13	-0.13	0.37	-0.06	-0.13	-0.03	0.00	-0.16	-0.02	-0.12	-0.07	-0.02	-0.19	-0.06	-0.05	-0.09	-0.13	-0.07
AT2	0.08	0.97	0.21	-0.15	-0.10	0.60	-0.06	-0.12	-0.03	-0.01	-0.11	-0.07	-0.13	-0.05	0.05	-0.21	-0.03	-0.06	-0.16	-0.16	-0.02
Aberrant Basaloid Cell	0.17	0.17	0.86	-0.15	-0.10	0.37	-0.13	0.01	0.27	-0.03	-0.15	-0.15	-0.20	-0.24	-0.12	-0.09	-0.09	-0.11	-0.09	-0.14	-0.04
Adventitial Fibroblast	-0.12	-0.17	-0.18	0.88	0.51	-0.20	-0.08	-0.12	-0.12	-0.09	-0.10	-0.17	-0.14	-0.22	-0.17	0.55	-0.10	0.04	-0.11	-0.04	-0.13
Alveolar Fibroblast	-0.11	-0.13	-0.19	0.49	0.90	-0.19	0.01	-0.14	-0.14	-0.08	-0.13	-0.13	-0.09	-0.17	-0.19	0.63	-0.13	-0.11	-0.06	-0.05	-0.15
Alveolar Intermediate	0.47	0.42	0.77	-0.21	-0.18	0.74	-0.13	-0.11	0.10	-0.01	-0.17	-0.13	-0.23	-0.20	-0.08	-0.23	-0.07	-0.09	-0.13	-0.21	-0.04
Alveolar Macrophage	-0.06	-0.05	-0.15	-0.11	-0.09	-0.12	0.92	0.10	-0.11	-0.03	0.13	-0.08	-0.11	-0.07	-0.14	-0.13	-0.08	-0.10	-0.02	0.70	-0.08
B	-0.08	-0.09	-0.17	-0.11	-0.08	-0.18	0.06	0.73	-0.14	-0.01	0.22	0.00	-0.08	-0.09	-0.13	-0.13	-0.13	-0.09	0.26	0.13	-0.13
Basal	-0.04	0.01	0.14	-0.14	-0.06	0.11	-0.09	-0.11	0.91	0.00	-0.05	-0.14	-0.13	-0.19	-0.17	-0.02	0.13	-0.13	-0.06	-0.10	0.17
Ciliated	-0.04	0.01	-0.03	-0.09	-0.12	0.01	-0.07	-0.04	0.12	0.96	0.00	-0.10	-0.12	-0.14	-0.16	-0.09	0.08	-0.07	-0.09	-0.04	0.09
DC	-0.15	-0.13	-0.22	-0.12	-0.09	-0.25	0.19	0.43	-0.15	-0.06	0.81	-0.06	-0.10	-0.03	-0.12	-0.11	-0.16	-0.10	0.21	0.36	-0.16
Aerocyte	-0.10	-0.11	-0.17	-0.14	-0.14	-0.19	-0.05	-0.01	-0.15	-0.07	-0.05	0.93	0.09	0.46	0.28	-0.15	-0.12	0.07	-0.10	-0.06	-0.14
Arterial	-0.19	-0.22	-0.28	0.11	0.21	-0.29	-0.08	-0.18	-0.24	-0.15	-0.17	0.14	0.79	0.49	0.33	0.24	-0.18	-0.04	-0.09	-0.11	-0.19
Capillary	-0.13	-0.14	-0.19	-0.21	-0.22	-0.20	-0.07	-0.14	-0.18	-0.11	-0.12	0.51	0.51	0.92	0.51	-0.19	-0.11	0.03	-0.14	-0.11	-0.15
Pulmonary Venous	-0.16	-0.18	-0.22	-0.14	-0.18	-0.26	-0.13	-0.19	-0.20	-0.14	-0.15	0.28	0.61	0.67	0.88	-0.15	-0.10	0.20	-0.12	-0.15	-0.14
Fibrotic Fibroblast	-0.17	-0.20	-0.18	0.51	0.59	-0.25	-0.06	-0.08	-0.14	-0.10	-0.05	-0.16	-0.02	-0.22	-0.14	0.79	-0.13	-0.09	-0.01	-0.01	-0.10
Goblet	-0.09	-0.03	-0.09	-0.19	-0.22	-0.04	-0.10	-0.10	0.20	0.01	-0.05	-0.17	-0.06	-0.18	-0.15	-0.18	0.94	-0.12	-0.12	-0.11	0.87
Lymphatic	-0.09	-0.11	-0.13	-0.03	-0.12	-0.14	-0.06	-0.09	-0.11	-0.07	-0.09	0.00	0.03	-0.02	0.16	-0.08	-0.09	0.96	-0.04	-0.06	-0.10
Mast	-0.05	-0.06	-0.13	-0.09	-0.12	-0.12	0.00	0.24	-0.11	-0.03	0.13	-0.06	-0.09	-0.11	-0.15	-0.12	-0.11	-0.13	0.80	0.05	-0.10
Monocyte	-0.08	-0.08	-0.18	-0.11	-0.11	-0.20	0.23	0.24	-0.16	-0.01	0.66	-0.08	-0.13	-0.07	-0.16	-0.12	-0.10	-0.13	0.22	0.66	-0.12
Secretory	0.08	0.17	0.33	-0.16	-0.22	0.64	-0.08	-0.15	0.34	0.05	-0.09	-0.22	-0.18	-0.26	-0.23	-0.19	0.34	-0.10	-0.15	-0.17	0.50
