# Supplementary material for: Alveolar niche disruption and aberrant epithelial reprogramming are early hallmarks of idiopathic pulmonary fibrosis
Source: bioRxiv. 2026 May 30:2026.05.27.727792. Preprint. [Version 1] doi: 10.64898/2026.05.27.727792 (PMC13317612; doi:10.64898/2026.05.27.727792)
Supplement: Supplement 3 [file media-3.pdf]

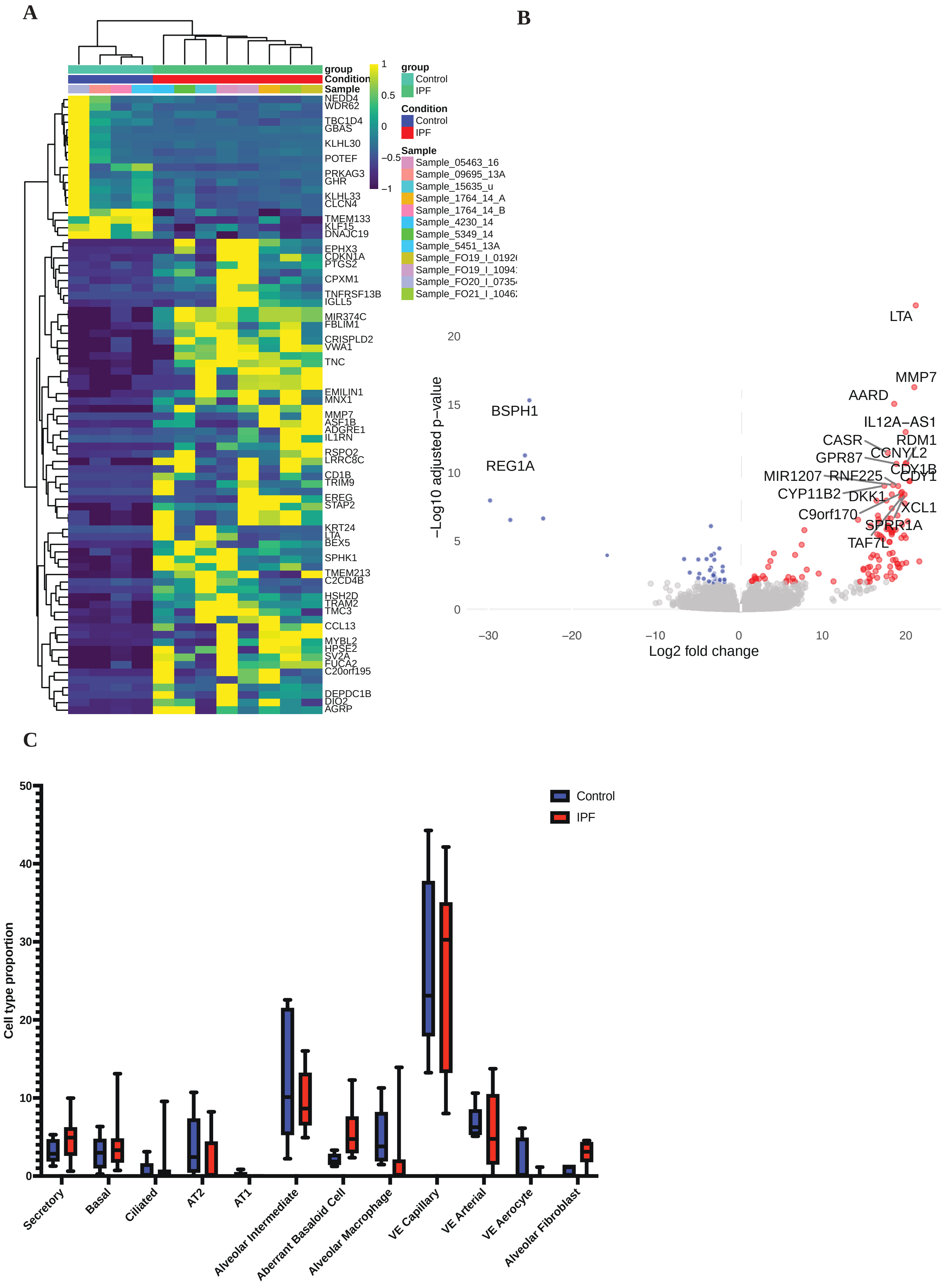

A
B
C
group
Condition
Sample
Control
IPF
Sample_05463_16
Sample_09695_13A
Sample_15635_u
Sample_1764_14_A
Sample_1764_14_B
Sample_4230_14
Sample_5349_14
Sample_5451_13A
NEDD4
WDR62
TBC1D4
GBAS
KLHL30
POTEF
PRKAG3
GHR
KLHL33
CLCN4
TMEM133
KLF15
DNAJC19
EPHX3
CDKN1A
PTGS2
CPXM1
TNFRSF13B
IGLL5
MIR374C
FBLIM1
CRISPLD2
VWA1
TNC
EMILIN1
MNX1
MMP7
ASF1B
ADGRE1
IL1RN
RSPO2
LRRC8C
CD1B
TRIM9
EREG
STAP2
KRT24
LTA
BEX5
SPHK1
TMEM213
C2CD4B
HSH2D
TRAM2
TMC3
CCL13
MYBL2
HPSE2
SV2A
FUCA2
C20orf195
DEPDC1B
DIO2
AGRP
−Log10 adjusted p−value
Log2 fold change
LTA
MMP7
AARD
BSPH1
IL12A−AS1
CASR
RDM1
GPR87
CCNYL2
REG1A
CDY1B
MIR1207
RNF225
CDY1
CYP11B2
DKK1
XCL1
C9orf170
SPRR1A
TAF7L
Cell type proportion
Control
IPF
Secretory
Basal
Ciliated
AT2
AT1
Alveolar Intermediate
Aberrant Basaloid Cell
Alveolar Macrophage
VE Capillary
VE Arterial
VE Aerocyte
Alveolar Fibroblast
